# A Retrospective Cohort Study in patients over 55: Mechanical versus Biological valve prostheses

**DOI:** 10.1186/1749-8090-10-S1-A345

**Published:** 2015-12-16

**Authors:** M Tarazi, K Sivabalah, N Mayooran, R Neagu, B Philip, MN Anjum, K Doddakula

**Affiliations:** 1 Department of Cardiothoracic Surgery, Cork University Hospital, Cork, Republic of Ireland

## Background/Introduction

The choice between a mechanical valve and a biological valve is not always clear for patients requiring valve replacement surgery; age has been considered to be the determining factor in the decision making process. Previous guidelines have recommended the implantation of mechanical prostheses in patients under the age of 65 and biological prostheses in those over 65.

## Aims/Objectives

To investigate the life expectancy of patients over 55 who have had either a bioprosthetic or mechanical valve replacement and to establish if there is a significant difference in survival rates between these two valve groups. In addition, this research aims to investigate if factors such as hypertension, diabetes and a history smoking have an impact on the patients' survival.

## Method

138 patient charts meeting the inclusion criteria, were identified using the percussionist database, and were used to extract the data relevant to the study. Data was analysed using SPSS.

## Results

Of the 138 patients, 59.8% (n = 61) of the patients with biological valves and 58.3% (n = 21) of the patients with mechanical survived between 5 to 10 years. The unpaired t-test and the chi-squared test performed showed that there was no statistically significant difference in the survival of patients who had biological valves and those who had mechanical valves.

## Discussion/Conclusion

This study suggests that the type of valve prosthesis implanted does not affect the survival of the patients.

